# Connecting the dots in translational bioinformatics: TBC 2014 collection

**DOI:** 10.1186/1755-8794-8-S2-I1

**Published:** 2015-05-29

**Authors:** Ju Han Kim

**Affiliations:** 1 Division of Biomedical Informatics, Systems Biomedical Informatics Research Center Seoul National University College of Medicine, 103 Daehak-ro, Jongno-gu, Seoul 110-799, Republic of Korea

## Introduction

The Translational Bioinformatics Conference (TBC) has been one of the most successful multi-disciplinary conferences in the rapidly emerging fields of bioinformatics and clinical genomics for their bidirectional translations. The Fourth Annual TBC 2014 jointly held with the 8^th ^International Conference on Systems Biology meeting for four days at the Huiquan Dynasty Hotel, Qingdao, China, improved our understanding of novel diagnostics and therapeutics in the era of biomedical big data.

While TBC is organized as an international forum for translational bioinformatics, the first three annual meetings of TBC have been held in Korea since 2011. We appreciate the Chinese Academy of Sciences for hosting TBC 2014 and making TBC a truly international one. Japanese Association of Medical Informatics (JAMI) has unanimously approved to host TBC 2015 in Tokyo in early November, 2015. TBC 2016 will either be held in India or United States. It is a great pleasure to see the real growth of TBC.

NIH Director Francis S. Collins said, "Data creation in today's research is exponentially more rapid than anything we anticipated even a decade ago." The ability to connecting the dots in the wealth biomedical big data will bring us the 'big picture' in a mass of genes, drugs, diseases, and diagnostic, therapeutic and prognostic markers. Steve Jobs said, "You can't connect the dots looking forward; you can only connect them looking backwards. So you have to trust that the dots will somehow connect in your future." Personalized medicine attempts to determine individual solutions based on the genomic and clinical profiles of each individual, providing opportunity to incorporate individual molecular data into patient care. While a plethora of genomic signatures have successfully demonstrated their predictive power, they are merely statistically-significant differences between dichotomized phenotypes that are in fact severely heterogeneous. Despite many translational barriers, connecting the molecular world to the clinical world and vice versa will undoubtedly benefit human health in the near future.

## Novel therapeutics and diagnostics markers for personalizing healthcare

Connecting experimental and/or observational data with factual bio-databases and biomedical literatures is an essential step in the course of translational bioinformatics analysis. Grover et al. (Deakin U., Australia) applied Gentrepid [1], a candidate gene prediction method empowered by five bioinformatics modules, to reanalyze Welcome Trust Case-Control Consortium GWAS data for coronary artery disease and successfully replicated 55% of the candidate genes identified by CARDIoGRAMplusC4D consortium meta-analysis [2]. By integrating the predicted candidate genes with the Therapeutic Target Database, PharmGKB, and DrugBank, they were able to identify highly-validated novel therapeutics feasible for repositioning as well as therapeutic target genes. Connecting drug-gene-disease associations is further boosted by adding protein complex information by Yu et al. (Xidan U., China) [3] who obtained indirect weighted bipartite relationships between drugs and diseases from the tripartite drug-gene-disease network. They performed two case studies for mental disorders and hypertension and successfully validated their network with comparative toxicogenomics database (http://ctdbase.org). Zhu et al. (Wuhan U.) integrated gene-expression prognostic markers for breast-cancer survival from Gene Expression Omnibus (http://www.ncbi.nlm.nih.gov/geo/) with drug sensitivity data extracted from the Developmental Therapeutic Program database (http://dtp.nci.nih.gov/). They were able to suggest a few repositioned drugs for breast cancer [4].

Despite of comprehensive databases and bioinformatics tools for analyzing genetic variants, genome interpretation at the personal level still remains a challenging goal. Na et al. (Seoul National U., Korea) proposed a computational scheme to connect individual personal genomes to disease predispositions for the purpose of personal genome interpretation [5]. Given a personal genome, they computed functional impacts of all potentially damaging missense variants by using the Sorting Intolerant From Tolerant (SIFT) algorithm [6]. Disease-gene links were obtained from the Online Mendelian Inheritance in Man (OMIM) by simultaneously considering the hierarchical structure of MeSH (Medical Subject Headings) terms. Similarity structure analysis with all-pairwise computation of mutual information between the SIFT-score vectors of variants of the personal genomes and the disease-gene association-score vectors revealed the connections between personal genomes and diseases.

Connecting the dots can be corrected in a relevant manner looking backward. While cancer cell lines have been extensively used for cancer research, measures for the similarity between cell lines and tumors are not fully established. Chen et al. connected 200 hepatocellular carcinoma (HCC) tumor samples from the The Cancer Genome Atlas and over 1000 cancer cell lines by using gene expression data [7]. While the most commonly used HCC cell lines resembled primary HCC tumors, nearly half of the cell lines did not. Selection of cancer cell lines may be benefited by the relevance measures of specific genes under investigation between the dots.

## Translational epigenomics

Connecting a variety of epigenomic mechanisms including histone modifications, post-transcriptional modifications (PTMs), and RNA editings to genes, drugs, and diseases, for investigating epigenomic regulations needs much more work to be done by translational bioinformatics researchers. Yang et al. (Chicago U., U.S.A.) proposed a computational scheme to integrate tissue-specific histone modifications and genome-wide transcriptional regulation [8]. While therapy-related, secondary acute myeloid leukemia (t-AML) has been suggested to be related to the suppression of a histone methyltransferase, EZH2, the critical target genes of EZH2 and their regulatory roles are largely unknown. Yang et al. developed the 'seq2gene' algorithm to explore target genes of immuneprecipitation sequencing (ChIP-seq) enriched regions and then extracted regulatory 'biomodules' enriched with genes with similar expression profile and genomic or functional characteristics by combining the seq2gene algorithm with Phenotype-Genotype-Network (PGnet) algorithm [9]. This preliminary analysis suggested SEMA3A (Semaphoring 3A) as a novel oncogenic candidate that is regulated by EZH2 silencing and warranted further study.

Altered PTM sites may be resulted by non-synonymous SNPs (nsSNPs) in the coding regions that are disease-associated. Kim et al (KAIST, Korea) created an open-access PTM-SNP database for comprehensive collection of human SNPs that affect PTM sites together with human disease associations extracted from GWAS catalogs [10]. They found that PTM-SNPs are highly enriched with human disease-associated nsSNPs. Post-transcriptional sequence modification of transcripts through RNA editing is also an important mechanism for regulating protein function and is associated with many human diseases. Lee et al. (Seoul National U., Korea) created RCARE (RNA-Seq Comparison and Annotation for RNA Editing) for searching, annotating, and visualizing RNA-DNA difference (RDD) sites [11]. RCARE as an open-access toolkit tries to manage problematic false positives, determine the location of condition-specific RDD sites, and elucidate their functional roles with evidence levels, summary plots and executive summary.

## Translating disease networks and network biomarkers

Despite the plethora of high-throughput data and the recent advances in next-generation sequencing technologies, correctly connecting the multi-level biomedical networks remains challenging. Network-based analysis of public databases and numerous biomedical knowledge resources are invaluable for a high profile translational bioinformatics research. Carson et al (U. of Illinois at Chicago) investigated human protein interaction network using the Disease Ontology in an attempt to identify disease-associated vs. non-disease-associated proteins [12]. Using a bootstrapping method, they created and trained an alternating decision tree classifier to extract conserved characteristics shared by disease-associated proteins with 79% area under the receiver operating curve. A variety of network properties and first- and second-order neighbours in the protein interaction network could improve the overall performance. To overcome the classical gene biomarker detection methods based on differentially expressed genes (DEGs) in studies with small number of samples, resulting too many false positives and low statistical power, Hur et al. (Seoul National U., Korea) proposed a multi-step filtering method to predict gene biomarkers from RNA-Seq data of case-control mouse-knockout studies [13]. They devised four-step filtering methods gradually combining DEG fold change, gene regulatory network membership, biological pathway membership, and single nucleotide variant frequency filters, to carefully reduce candidate gene biomarkers. Rather than detecting individual molecular biomarkers, Xin et al. (Chinese Academy of Sciences, China) developed a method to detect biomarkers at a network level based on protein-protein interaction affinity (PPIA) network modelling using linear programming [14].

## Methods for high performance translation

Better bioinformatics tools and methods are required for a successful translational research. In this issue, advanced solutions for well-known bioinformatics problems were introduced. Sandhan et al. (Seoul National U., Korea) proposed a protein function prediction method [15]. To overcome classical prediction methods that rely mainly on strong global features and sequence homologies, they constructed protein-protein similarity network that considers both global and local features, by capturing weakly-interacting pairs and by using the hierarchical voting algorithm via the graph pyramid. Wang et al. (Xiamen U., China) proposed *SeedsGraph*, a new de novo sequence assembly algorithm for whole-genome shotgun assembly in a cloud computing framework [16]. The MapReduce framework is used for the first sequence-read overlap step for short reads to reduce computational cost. The overlap graphs are then clustered into groups and compressed into chains of seeds that are used to construct a seeds graph by seeds overlapping. *PDEGEM *(Positional Dependent Energy Guided Expression Model) is proposed as a novel algorithm for estimating transcript abundances for RNA-Seq data by Xia et al. (Peking U., China) [17] based on Positional Dependent Nearest Neighbourhood model. Hayashida et al. (Kyoto U., Japan) introduced three parallelized algorithms, *BfsEnumP1-3*, by modifying their previous algorithm, *BfsSimEnum*, for enumerating tree-like chemical compounds without multiple bonds [18]. Enumerating chemical compound is essential in designing and finding new drugs and determining chemical structures from mass spectrometry data. By dividing a set of vertices into several subsets and assigning them to microprocessors, *BfsEnumP1-3 *greatly reduced execution time with high parallelization efficiency.

This meeting, TBC 2014, provided an international forum for translational bioinformatics and clinical genomics researchers to bring together and substantially improve our understanding of molecular and pathophysiologic foundations of human diseases and health. I congratulate the speakers and authors to this conference who are shaping the future of personalized diagnostic, prognostics and therapeutics. Today, many health topics for personalized and precision medicine are increasingly within the scope of translational bioinformatics. It would be fascinating for our generation to see the transformation of traditional trial-and-error medicine into informatically-empowered personalized-and-precision medicine.

## Competing interests

The author declares that they have no competing interests.

## Declarations

TBC 2014 and the publication costs of this research was supported by Basic Science Research Program through the National Research Foundation of Korea (NRF) grant funded by the Korea government (MSIP) (2013-005540) and by a grant of the Korean Health Technology R&D Project, Ministry of Health and Welfare (HI13C2164).

This article has been published as part of *BMC Medical Genomics *Volume 8 Supplement 2, 2015: Selected articles from the 4th Translational Bioinformatics Conference and the 8th International Conference on Systems Biology (TBC/ISB 2014). The full contents of the supplement are available online at http://www.biomedcentral.com/bmcmedgenomics/supplements/8/S2.

## References

[B1] Ballouz S Liu J Oti M Gaeta B Fatkin D Bahlo M Wouters M Analysis of genome-wide association study data using the protein knowledge base BMC Genet 2011 12 98 22077927 10.1186/1471-2156-12-98 PMC3261104

[B2] Grover MP Ballouz S Wouters M Novel therapeutics for coronary artery disease from genome-wide association study data BMC Med Genomics 2015 Suppl S 10.1186/1755-8794-8-S2-S1 PMC4460746 26044129

[B3] Yu L Huang J Ma Z Zhang J Zou Y Gao L Inferring drug-disease associations based on known protein complexes BMC Med Genomics 2015 Suppl S 10.1186/1755-8794-8-S2-S2 PMC4460611 26044949

[B4] Zhu L Zhu F Identification association of drug-disease by using functional gene module for breast cancer BMC Med Genomics 2015 Suppl S 10.1186/1755-8794-8-S2-S3 PMC4460962 26045063

[B5] Na YJ Sohn KA Kim JH Interpretation of personal genome sequencing data in terms of disease ranks based on mutual information BMC Med Genomics 2015 Suppl S 10.1186/1755-8794-8-S2-S4 PMC4460593 26045178

[B6] Kumar P Henikoff S Ng PC Predicting the effects of coding non-synonymous variants on protein function using the SIFT algorithm Nat Protocols 2009 4 7 1703 1081 10.1038/nprot.2009.86 19561590

[B7] Chen B Sirota M Fan-Minogue H Hadley D Butte A Relating Hepatocellular Carcinoma Tumor Samples and Cell Lines Using Gene Expression Data in Translational Research BMC Med Genomics 2015 Suppl S 10.1186/1755-8794-8-S2-S5 PMC4460709 26043652

[B8] Yang X Wang B Cunningham J Identification of epigenetic modifications that contribute to pathogenesis in therapy-related AML: Effective integration of genome-wide histone modification with transcriptional profiles BMC Med Genomics 2015 Suppl S 10.1186/1755-8794-8-S2-S6 PMC4460748 26043758

[B9] Yang X Huang Y Chen JL Xie J Sun X Lussier YA Mechanism-anchored profiling derived from epigenetic networks predicts outcome in acute lymphoblastic leukemia BMC Bioinformatics 2009 10 Suppl 9 S6 10.1186/1471-2105-10-S9-S6 19761576 PMC2745693

[B10] Kim Y Kang C Min B Yi GS Detection and Analysis of Disease-associated Single Nucleotide Polymorphism Influencing Post-translational Modification BMC Med Genomics 2015 Suppl S 10.1186/1755-8794-8-S2-S7 PMC4460713 26043787

[B11] Lee SY Joung JG Park CH Park JH Kim JH RCARE: RNA Sequence Comparison and Annotation for RNA Editing BMC Med Genomics 2015 Suppl S 10.1186/1755-8794-8-S2-S8 PMC4460956 26043858

[B12] Carson M Lu H Network-based Prediction and Knowledge Mining Of Disease Genes BMC Med Genomics 2015 Suppl S 10.1186/1755-8794-8-S2-S9 PMC4460923 26043920

[B13] Hur B Chae H Kim S Combined analysis of gene regulatory network and SNP information enhances identification of potential gene markers in mouse knockout studies with small number of samples BMC Med Genomics 2015 Suppl S 10.1186/1755-8794-8-S2-S10 PMC4460612 26044212

[B14] Xin J Ren X Chen L Wang Y Identifying network biomarkers by protein-protein interaction affinity derived from law of mass action BMC Med Genomics 2015 Suppl S 10.1186/1755-8794-8-S2-S11 PMC4460625 26044366

[B15] Sandhan T Yoo Y Choi JY Kim S Graph Pyramid Approach for Protein Classification BMC Med Genomics 2015 Suppl S

[B16] Wang C Guo M Liu X Liu Y Zou Q SeedsGraph: an efficient assembler for next generation sequencing data BMC Med Genomics 2015 Suppl S 10.1186/1755-8794-8-S2-S13 PMC4460749 26044652

[B17] Xia Y Wang F Qian M Qin Z Deng M PDEGEM: Modeling non-uniform read distribution in RNA-seq data BMC Med Genomics 2015 Suppl S 10.1186/1755-8794-8-S2-S14 PMC4460722 26044773

[B18] Hayashida M Jindalertudomdee J Zhao Y Akutsu T Parallelization of Enumerating Tree-like Chemical Compounds by Breadth-first Search Order BMC Med Genomics 2015 Suppl S 10.1186/1755-8794-8-S2-S15 PMC4460618 26044861

[B19] Gardeux V Arslan AD Achour I Ho TT Beck WT Lussier YA Concordance of deregulated mechanisms unveiled in underpowered experiments: PTBP1 knockdown case study BMC Med Genomics 2014 Suppl S 10.1186/1755-8794-7-S1-S1 PMC4101571 25079003

